# Exosomal circRNAs: Sorting Mechanisms, Roles and Clinical Applications in Tumors

**DOI:** 10.3389/fcell.2020.581558

**Published:** 2020-11-25

**Authors:** Yanhua Xu, Shan Kong, Shiyi Qin, Xianjuan Shen, Shaoqing Ju

**Affiliations:** ^1^Department of Laboratory Medicine, Affiliated Hospital of Nantong University, Nantong, China; ^2^Research Center of Clinical Medicine, Affiliated Hospital of Nantong University, Nantong, China; ^3^School of Medicine, Nantong University, Nantong, China

**Keywords:** exosomes, circRNAs, biomarkers, drug resistance, therapeutic targets, exosomal circRNAs

## Abstract

Exosomes are a group of nano-sized membrane vesicles and are important mediators of intercellular communication, particularly in tumor microenvironment. Recently, researchers have found that circular RNAs (circRNAs), with the great research significance, are enriched and stable in exosomes. In this review, we summarize the research significance of exosomal circRNAs, sorting mechanisms and their functioning mechanisms in tumor progression. Their clinical applications as clinical tumor biomarkers and as therapeutic targets in inhibiting tumor metastasis, anti-cancer immunity response and drug resistance have been widely discussed.

## Introduction

Exosomes were first identified in sheep reticulocytes by Harding et al. (1983) in 1983 and were named “exosomes” by Pan and Johnstone (1983) 4 years later. The role of exosomes was then speculated to be a way for cells to excrete waste. In 1996, Raposo et al. (1996) found that immune cells which similar to B lymphocytes also secrete antigen-presenting vesicles, which can directly stimulate the anti-tumor response of CD4+ cells. In 2007, Valadi et al. (2007) further discovered that cells could exchange genetic material through RNA in exosomes. With the deepening of the research, researchers found exosomes are widely involved in various biological processes such as immune response, antigen presentation, cell differentiation, tumor growth and invasion (Ibrahim and Marbán, 2016). Exosomes are small membrane vesicles with lipid bilayer structure, approximately 40–160 nm in diameter (Kalluri and LeBleu, 2020). Studies have shown that specific molecular markers such as lipids, proteins and RNA exist on the surface of exosomes. They also contain various components inside, such as RNA, proteins and even DNA, which are transported to the recipient cells to come into play (Bebelman et al., 2018). Because of these advantages, exosomes play crucial roles in a variety of diseases, especially mediating the communication between tumor cells.

Following microRNA (miRNA) and long non-coding RNA (LncRNA), circRNA is an emerging endogenous non-coding RNA (ncRNA) with excellent research potential, which is a new member of the non-coding RNA family. In 1976, Sanger (Sanger et al., 1976) first found covalently closed circRNAs in plant viroids, and in 1979, when Hsu (Hsu and Coca-Prados, 1979) found similar circRNAs in the cytoplasm of HeLa cells by electron microscopy, it was believed that circRNAs were the product of faulty splicing (Cocquerelle et al., 1993). Until 1993, Capel (Capel et al., 1993) found that circular RNA genes of mice Sry (sex-determining region Y) may exert a specific function in mice testicles, which truly brought circular RNA into the field of scientific research vision, and gradually become the focus of research. In recent years, with the development of RNA research technology, researchers have found a large number of circRNAs in diverse organisms. These circRNAs have important biological functions, such as serving as miRNA sponges, interacting with RNA binding proteins (RBPs), and encoding proteins or peptides (Hansen et al., 2013; Zheng et al., 2016; Hsiao et al., 2017). Previous studies have shown that circRNAs are closely associated with diabetes (Zhu et al., 2019), neurological diseases (You et al., 2015), cardiovascular diseases (Zhang S. et al., 2020), and cancer (Zhang N. et al., 2020), and also play an important role in the early diagnosis and clinical treatment of diseases (Xian et al., 2020). CircRNAs are expected to be extracted from samples and analyzed, so studies on their potential as molecular markers are being undertaken widely.

Intriguingly, recent research found that the expression of circRNAs in exosomes is abundant and stable. Exosomal circRNAs can play a significant role after being absorbed by recipient cells (Li et al., 2015). In this review, we elucidate how circRNAs involved in exosomes and the mechanisms by which exosomal circRNAs play a role in tumors. We particularly emphasize the mechanism of mediating tumor drug resistance and their clinical applications in tumor therapy.

## Current Research Situation of Exosomal CircRNAs

### Biogenesis and Characteristics of circRNAs

**FIGURE 1:**
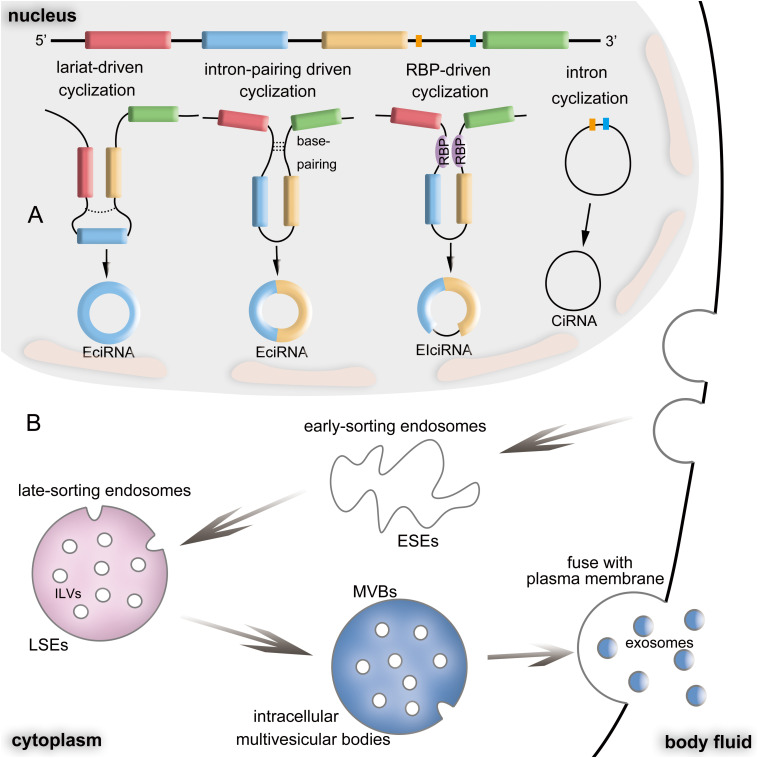
Biogenesis of circRNAs and exosomes. **(A)** The biogenesis of circRNAs: The mechanisms of lariat-driven cyclization, intron-pairing driven cyclization, RBP-driven cyclization and intron cyclization can form EcRNAs, EIciRNAs and CiRNAs. **(B)** The biogenesis of exosomes.

CircRNAs are generated from specific alternative splicing of pre-mRNA (Fu and Ares, 2014) and can be divided into three categories according to its components: Exon circRNAs (EciRNAs), Intron circRNA (CiRNAs), and Exon-Intron circRNA (EIciRNAs) (Belousova et al., 2018; Wang and Fang, 2018). Currently, two mechanisms for CircRNA formation are widely accepted: (1) Back-splicing hypothesis is that the downstream splicing site is reversely connected to the upstream splicing site to form a closed circRNA molecule (Ebbesen et al., 2017). This cyclization is mainly caused by base-pairings between repeating elements in the upstream or downstream introns (Ivanov et al., 2015; Kelly et al., 2015), or by the dimerization of RBPs (Conn et al., 2015), or by the combination of RBPs with the motif in the flanking introns (Zhang et al., 2014; Errichelli et al., 2017), which are commonly referred to as intron-pairing-driven cyclization (Ebbesen et al., 2017; Chuang et al., 2018; Eger et al., 2018; Liu et al., 2019) and RBP-driven cyclization (Conn et al., 2015). This mechanism mainly produces EciRNAs and EIciRNAs; (2) Lariat-driven cyclization formed by exon-skipping reading is another mechanism of circRNAs formation (Eger et al., 2018). Besides, circRNAs are also produced by intron lariat detaching from the branching structures during splicing, which leads to the formation of EciRNAs and CiRNAs (Zhang et al., 2013; Lasda and Parker, 2014; Eger et al., 2018). Mechanisms for circRNAs’ formation are shown in (Figure 1A).

Compared to other ncRNAs, circRNAs have some unique characteristics. Firstly, there are thousands of tissue-specific circRNAs in eukaryotes (Wang et al., 2014; Westholm et al., 2014; Ivanov et al., 2015). For example, circRNAs from Rmst and Klhl2 were highly expressed in the brains of mice, but not in the liver or lungs (You et al., 2015). In drosophila, circMbl was far less expressed in the ovaries than in the head (Westholm et al., 2014). Secondly, due to its unique closed-loop structure, circRNAs have good stability, long half-life (Enuka et al., 2016), and are hard to be degraded by exonuclease (Jeck et al., 2013; Memczak et al., 2013).

### Formation and Release of Exosomes

Exosomes are a class of extracellular vesicles (EVs), which are membrane vesicles secreted by various types of cells. Compared with other EVs, exosomes have unique ways of formation and release: the cytoplasmic membrane is enmeshed, and some extracellular components and cell membrane proteins are wrapped together to form early-sorting endosome (ESEs), which can exchange materials with other organelles, or fuse with different ESEs to form late-sorting endosomes (LSEs), and then further form into intracellular multivesicular bodies (MVBs), which contain many intraluminal vesicles (ILVs) (Mathieu et al., 2019; McAndrews and Kalluri, 2019). During the process of forming endosomal membranes, the cytoplasmic RNA molecules (mRNA and miRNA) and some functional proteins are wrapped up in it (Kulkarni et al., 2019). ILVs, which were broadly considered to be exosomes, can be released by the fusion of MVBs with the plasma membrane (PM) into the extracellular space (Kowal et al., 2014; Frydrychowicz et al., 2015; Bebelman et al., 2018). MVBs may also be degraded by fusion with autophagosomes or lysosomes to release ILVs as exosomes (Kahlert and Kalluri, 2013; van Niel et al., 2018). Unique formation and release way of exosomes are shown in Figure 1B. After being released, exosomes may exist around the releasing cell or remain in the extracellular space. Additionally, they can move dynamically in body fluids. Exosomes can also be absorbed by adjacent and distant cells, thus changing the behaviors of target cells (Melo et al., 2014). However, the absorption of exosomes is not a random event. Some reports have explained that several mediators with specific roles can help exosomes find their target cells, including lipids, proteins, and some extracellular matrix. For example, cancer-derived exosomes can target specific tissues, such as the liver and lung, and promote the formation of a premetastatic niche by the composition of integrin (Hoshino et al., 2015). In addition, some cell-derived exosomes themselves can expose adhesion molecules on their surfaces to attract and grab target cells (Théry et al., 2009).

### Discovery of Exosomal circRNAs

Exosomes contain many RNAs, including mRNA and ncRNAs (Skog et al., 2008; Taylor and Gercel-Taylor, 2008; Lai et al., 2010). Surprisingly, Li et al. (2015) found circRNAs were abundant and stable in exosomes from MHCC-LM3 HCC cells through RNA-seq analysis. Their results indicated that exosomes contained at least twice as many circRNAs as the circRNA-producing cells themselves. In addition, the genome-wide analysis revealed that the ratio of circRNAs to linear RNA in exosomes was 6-8 times than that of the producing cells, and more than 1,000 different circRNAs have been found in exosomes of human serum (Bao et al., 2016).

## Possible Mechanism of circRNAs Sorting Into Exosomes

Although RNA loading is a random event, different levels of RNA enrichment in EVs suggest that RNA sorting is regulated by specific mechanisms (Koppers-Lalic et al., 2014; Hinger et al., 2018). The mechanism often relies on RBPs and their relative partners, which can target RNAs to the site of EV generation and protect them from degradation. In recent years, researchers have been exploring how RNAs load into EVs. MiRNAs are the most widely studied. Using qPCR, the researchers found that when the expression level of miRNAs in cells changed, the corresponding miRNAs expression level in exosomes would also show the same change trend, but with a larger change range. It was further found that the sorting of miRNAs into exosomes is directly regulated by the abundance of their targeted mRNAs in the cells that produce these miRNAs. When the targeted mRNAs are increased, the miRNA is more likely to remain in the cell and be excluded from the exosomes. Conversely, if mRNA levels decrease, the miRNAs is loaded into the exosomes and secreted (Squadrito et al., 2014). Second, Ago2, the main components of miRISCs (miRNA loaded RNA-induced silencing complexes), may serve as an important transferring machinery for EV-miRNAs (McKenzie et al., 2016). Knockdown of Ago2 reduces several miRNAs sorting to EVs. Besides, endosomal sorting complex required for transport (ESCRT) proteins, such as Alix and Aps4A, have been shown to regulate miRNA sorting to EVs (Kosaka et al., 2010; Wei et al., 2015; Iavello et al., 2016). Third, some RBPs were shown to mediate specific miRNAs to EVs (Teng et al., 2017; Clancy et al., 2019). There still remains certain RBPs which perform miRNAs sorting by recognizing specific RNA motifs (Villarroya-Beltri et al., 2013; Lee et al., 2019). For example, hnRNPA2B1 can bind and convey miRNAs with GGAG motif into exosomes. HnRNPA2B1 was also reported to regulate the loading of lncARSR into exosomes, which depends on the specific sequence at the 5′ end (Qu et al., 2016).

Although there is no clear evidence to show how lncRNAs target EV-producing sites, they may share the similar sorting mechanisms with mRNAs (Batagov et al., 2011). MRNAs have been shown to differentially sorted to EVs mostly depending on their specific sequences and secondary structures in the 3′-untranslated regions. For example, lncRNA YBX1 could participate in the sorting process by specifically binding with corresponding motifs (Kossinova et al., 2017; Yanshina et al., 2018).

Considering that the types of circRNAs in exosomes are different from those in multiple cell lines, the mechanism of circRNAs entering exosomes deserves further investigation. Research showed that the abundance of exo-circRNAs was only moderately correlated with the level of cellular circRNAs, indicating that the sorting of specific circRNA species to exosomes may be actively regulated (Bao et al., 2016). Since circRNAs have been reported to bind to miRNAs in cells, and miRNAs are also abundant in exosomes, Li et al. (2015) speculated that there may be a relation between miRNAs and circRNAs in producer cells in the sorting of circRNAs into exosomes. The results suggested that the sorting of circRNAs to exosomes was at least, to some extent, regulated by the changes of related miRNAs levels in producer cells. For example, the authors introduced miR-7 mimics into cells and measured the abundance of CDR1as/cirS-7, the results revealed that the abundance of CDR1as/cirS-7 decreased in exosomes compared with that in the producer cells. Meanwhile, circRNAs still retain their original biological functions in receptor cells. In previous studies, Dou et al. (2016) explored 7 circRNAs with the highest abundance in exosomes of DKs-8 cells were also high expressed in DKs-8 cells, indicating that some circRNAs in the cells could be transferred into exosomes. However, RT-PCR showed that circRTN4 was high expressed in the exosomes of DLD-1 cells and low in DLD-1 cells, which indicated that molecular transport between cells and their exosomes was very complex.

Besides, other possible mechanisms include RNA-associated proteins binding to circRNAs (Bao et al., 2016). Nonetheless, the precise mechanism of circRNA sorting remains largely unknown.

## Regulatory Mechanisms of Exosomal circRNAs in Tumors

### Exosomal circRNAs Act as miRNA Sponges to Exert Biological Functions

**FIGURE 2:**
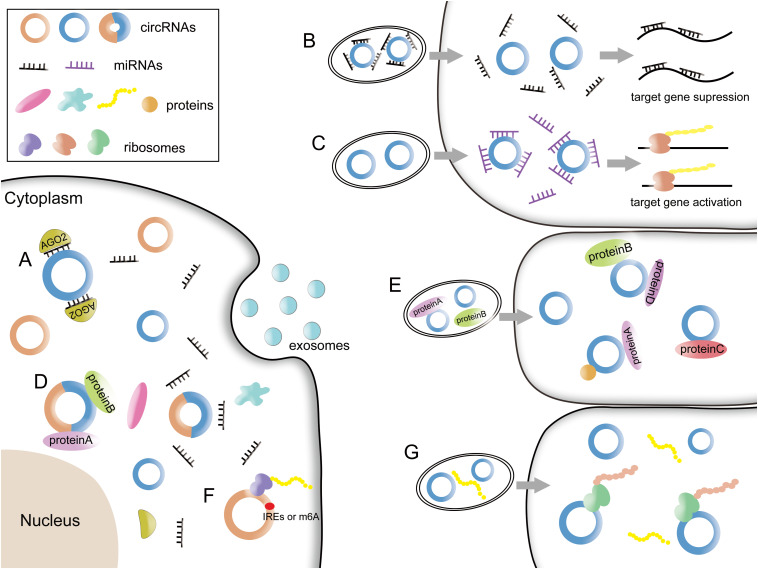
Functions of circRNAs and exosomal circRNAs. **(A)** CircRNAs affect the development of diseases by competitively binding to miRNAs. **(B)** Some exoxomal circRNAs bind to miRNAs in exosomes. After being released, miRNAs detach from circRNAs and silence the target genes in recipient cells. **(C)** Upon arriving, circRNAs can bind to miRNAs which exist in the recipient cells to activate the target genes. **(D)** CircRNAs can interact with RBPs. **(E)** Some exosomal circRNAs may bind to proteins in exosomes and be transported together to target cells. Or they can be transferred to recipient cells and then combine with proteins to come into effect. **(F)** CircRNAs with IREs or m6A modification can encode proteins or peptides. **(G)** Exosomes may transmit circRNAs to recipient cells to encode proteins or peptides to regulate the activity of life. The functional proteins may also be transported to recipient cells.

MiRNAs are a class of endogenous, regulatory ncRNAs with a length of about 20–25 nucleotides in eukaryotes (Macfarlane and Murphy, 2010). MiRNAs can identify mRNAs by complementary pairings, guide the RISCs to degrade mRNAs or inhibit the translation of mRNAs (Macfarlane and Murphy, 2010). CircRNAs have abundant miRNA binding sites. Large numbers of studies have shown that circRNAs act as miRNA sponges, thereby eliminating the inhibition of miRNA on target genes and increasing the expression level of target genes (Figure 2A; Denzler et al., 2014; Thomson and Dinger, 2016).

Studies have shown that exosome-derived circRNAs can still function as miRNA sponges after targeting recipient cells. The transfer of exosomes makes the circRNAs in exosomes more flexible and generally regulate the downstream target genes. There may be two situations: (1) CircRNAs bind with miRNAs and enter the exosomes together. Upon arrival at the recipient cells, miRNAs detach from circRNAs and then bind to the mRNAs in the recipient cell, thereby silencing mRNAs (Figure 2B). (2) Another assumption is that after circRNAs enter the recipient cells through exosomes, they absorb miRNAs in recipient cells to alleviate the inhibitory effect of miRNAs on the corresponding mRNA (Figure 2C).

The latter has found the following main types so far:

1. Target at adipocytes: (Zhang et al., 2019b) found that the exosomal ciRS-133 in the plasma of gastric cancer (GC) patients was up-regulated, and in vivo experiments showed that the ciRS-133 secreted by GC cells could be transferred into the pre-adipocytes by exosomes, thereby promoting the expression of Positive Regulatory Domain containing 16 (PRDM16), a key factor determining the fate of adipocyte differentiation, by inhibiting the biological function of miR-133. Upregulation of PRDM16 in preadipocytes determined the direction of cell proliferation and activated the expression of UCP1, so this study determined that circRNAs produced by cancer cells play an important role in the regulation of WAT Browning and tumor-related cachexia through exosomes.
2. Target at normal liver cells (L-02): (Dai et al., 2018) found that circRNA_100284 was up-regulated in arsenite-transformed L-02 cells, and their exosomes could be absorbed by normal L-02 cells, resulting in the up-regulated expression of circRNA_100284 in normal liver cells. Subsequently, they showed that circRNA_100284, delivered by exosomes to normal liver cells, could up-regulate the expression of Enhancer Of Zeste Homolog2 (EZH2) which is a potential marker for proliferation and cyclin-D1 which regulates the G1 to S phase transition in the cell cycle by acting as a sponge for miR-217, thus accelerating the cell cycle, promoting cell proliferation and malignant transformation of untransformed cells in hepatocellular carcinoma (HCC). Besides, the researchers found that circRNA Cdr1as is highly expressed in HCC patients cell lines and their exosomes, and can accelerate the development of cancer through miR-1270 sponge action. In HCC cell lines, the expression of circRNA Cdr1as was significantly higher than that of surrounding normal 293T cells. Exosomes secreted by HCC cells can transfer circRNA Cdr1as to surrounding normal cells, increasing the content of circRNA Cdr1as in 293T cells, thereby significantly enhancing the proliferation and migration ability of 293T cells (Su et al., 2019).
3. Target at human microvascular vein endothelial cells (HUVECs): HUVECs is a barrier that controls the exchange of substances between surrounding tissues and blood, it is an important factor to prevent the invasion of pancreatic carcinoma (PADC) cells. Studies have shown that exosomes from PADC cells transferred circ-IARS to HUVECs, which could absorb miR-122 and remove its inhibition on target gene RhoA and activate the RhoA signaling pathway, thus increasing endothelial monolayer permeability and promoting invasion and metastasis of PADC (Li J. et al., 2018).
4. Target at tumor cells: in addition to affecting normal cells in the body, exosomal circRNAs secreted by tumor cells also can perform effects on the same type of cancer cells with low metastatic ability. For example, Wang G. et al. (2019) conducted further studies on the interactions between HCC cells with non-metastatic (HepG2), low metastatic (97L) and high metastatic ability (LM3). The results showed that exosomes released by highly metastatic HCC cells with a high abundance of circPTGR1 could down-regulate the expression of miR449a in recipient cells and thus promoted the expression of MET, enhance the migration and invasion of HCC cells with low or no metastasis, and lead to destruction of the tumor microenvironment homeostasis and promote HCC progression.

Recent researches have shown that normal cells can also transmit circRNAs to tumor cells through exosomes and function as sponges, thus promoting or inhibiting tumor progression in recipient cells. For example, Zhang et al. (2019a) found that the expression of circ-DB in exosomes secreted by adipocytes is higher in patients with higher body fat rate. Circ-DB can absorb miR-34a to promote the expression of Ubiquitin-specific protease 7 (USP7) and Cyclin A2. At the same time, they also found that the exosomes secreted by adipocytes can promote the proliferation and reduce DNA damage in HCC. Experimental data in vivo showed that exosomes secreted by adipose tissues could significantly reduce the level of miR-34a and activate the USP7/Cyclin A2 pathway. However, effects all the above could be eliminated after the knocking down of circ-DB. Thereby, it can be concluded that adipose-cell-derived exosomes could mediate the transmission of circ-DB and promote the development of HCC by regulating the ubiquitin-related miR-34a/USP7 pathway. In addition, Chen et al. (2020) detected exosomal circRNAs in plasma by microarray sequencing and found that the expression of circ-0051443 was significantly reduced in both exosomes of HCC tissues and plasma. In this study, it was found that normal cells could deliver specific circ-0051443 to HCC cells via exosomes. When exosomes were absorbed by HCC cells, circ-0051443 bound to miR-331-3p competitively and reduced the expression of Bcl2 Antagonist/Killer 1 (BAK1) which is an important cell death regulator, thereby promoting apoptosis of HCC cells and inhibiting cell cycle to control the progression of malignant tumors.

Exosomes can gather circRNAs within vesicles with an abundance of 2–4 times greater than that in the cytoplasm and transport them to target cells (Li et al., 2015). With the targeting effect of exosomes, circRNAs can not only promote or inhibit cancer in a cell or a piece of tissue but also can be delivered to adjacent or distant cells or tissues to play a role as a potential therapeutic target for tumors.

### Exosomal circRNAs Interact With Binding Proteins to Exert Biological Functions

RNA binding proteins are a group of proteins that bind to RNA and regulate the metabolism. More than 800 RBPs have been identified in the human genome (Lunde et al., 2007). They can accompany the whole life of circRNAs, including production, post-transcriptional regulation, translation, functional execution and potential extracellular transport pathways (Zang et al., 2020). The interactions between circRNAs and RBPs are shown in Figure 2D.

Exosomal circRNAs can also interact with binding proteins in recipient cells to perform biological functions. Huang et al., 2020 found that circRNA-1,00,338 was significantly highly expressed in HCC cells and their exosomes, and the exosomal circRNA-1,00,338 could enhance the metastasis ability of HCC cells by enhancing the activity of angiogenic factors after entering recipient HUVECs. To further investigate the mechanism of exosomal circRNA-1,00,338, the authors transfected HUVECs with biotin-labeled circRNA-1,00,338 probes and performed RNA pull-down experiment. The results indicated that the RNA binding protein NOVA2, which regulates post-transcriptional modification of RNA, can bind to circRNA-1,00,338. NOVA2 has been reported to regulate vascular development and lumen formation (Giampietro et al., 2015), suggesting that the internalized exosomal circRNA-1,00,338 may regulate angiogenesis by interacting with the binding protein NOVA2. However, the authors didn’t confirm this with further experiments.

Another study suggests that exosomal circRNAs may play a biological role by interacting with RBPs in recipient cells. Firstly, researchers found that CD133^+^ cells isolated from colorectal cancer (CRC) cells could notably enhance cell stemness, sphere formation and metastasis. Next, they further cocultured CD133^–^ cells with exosomes from CD133^+^ cells, the results revealed that cell stemness, sphere formation and metastasis were remarkably enhanced. To further explore the mechanism of exosomes from CD133^+^ cells in CRC, researchers screened out the upregulated circ-ABCC1 which is a CRC-related circRNAs in exosomes by means of circRNADisease database. Naturally, researchers speculated that exosomes from CD133+ cells carrying circ-ABCC1 mediate cell stemness and metastasis in CRC. Subsequently, researchers inferred and preliminarily confirmed that circ-ABCC1 could bind with β−catenin into the cell nucleus and activate the Wnt pathway to regulate CRC progression via RNA Immunoprecipitation (RIP) and RNA pull-down experiments (Zhao et al., 2020). In this study, the researchers overexpressed circ-ABCC1 in recipient CD133^–^ cells to simulate exosome transporting circ-ABCC1 to recipient cells. However, this approach does have its drawbacks, the authors should further confirm whether exosomes secreted by CD133^+^ cells could transport circABCC1 to recipient CD133^–^ cells by some certain experiments. Under this condition, it can finally be concluded that exosomal circABCC1 does play a certain role in promoting CRC process via interacting with certain proteins.

Compared to the previous two studies, a recent study provided ample evidence that exosome-derived circRNAs can interact binding proteins and regulate tumor progression in recipient cells. Circ-CCAC1 expression was significantly increased in bile and serum extracellular vesicles in patients with cholangiocarcinoma (CCA), and the EVs of CCA cells carried circ-CCAC1 into vascular endothelial cells selectively. CCLP1 cell-derived EVs could significantly down-regulate the expression of ZO-1/Occludin in HUVEC, while the EVs overexpressed circ-CCAC1 in CCLP1 could significantly down-regulate the expression of Zo-1/Occludin. The RPISeq database predicted proteins that might interact with circ-CCAC1, and the prediction results indicated that EZH2, DNMT1, and STAU1 might interact with circ-CCAC1. The RIP and RNA pull-down experiments showed that there was an obvious interaction between EZH2 and circ-CCAC1. The interaction was also verified by fluorescence in situ hybridization (FISH) co-location analysis. Increased circ-CCAC1 in EVs significantly increased the cytoplasmic localization of EZH2, while decreased the expression of ZO-1 and Occludin. SH3GL2 is a negative regulator of ZO-1/Occludin, and circ-CCAC1 in the extracellular vesicle may increase the expression of SH3GL2. Chromatin Immunoprecipitation (ChIP) experiments showed that EZH2 could directly bind to the promoter of SH3GL2 and promote its H3K27me3 methylation level, and thus inhibit the expression of SH3GL2. Circ-CCAC1 derived from EVs could reduce the efficiency of EZH2 binding to the SH3GL2 promoter, thereby promoting the expression of SH3GL2, which further inhibited the expression of ZO-1/Occludin (Xu et al., 2020).

Therefore, we concluded that exosomal circRNAs also interact with binding proteins in recipient cells to exert biological functions. As is mentioned above, with the assistance of binding proteins, circRNAs enter the exosomes and are transported by exosomes to the recipient cells. Another situation is that circRNAs are transported by exosomes and released into the recipient cells, thus binding to the RBPs of the recipient cell to perform functions. More experiments are needed to verify these hypotheses. The potential interactions between exosomal circRNAs and RBPs are shown in Figure 2E.

### Possibility of Exosomal circRNAs Encoding Proteins or Peptides

Since circRNAs do not contain 5′caps, the translation is hat-independent. Some circRNAs have an internal ribosomal entry site (IRES) (Abe et al., 2015) or translate in an m6A-modified manner in the 5′ untranslatable region (UTR) (Meyer et al., 2015). While thousands of circRNAs are currently expected to contain a hypothetical open reading frame (ORF) and an upstream IRES, there are very few circRNAs that encode proteins or peptides actually. The pattern of circRNAs encoding proteins or peptides is shown in Figure 2F.

In glioblastoma, circ-SHPRH can be translated into SHPRH-146aa, which is involved in the occurrence and development of tumors of the central nervous system by regulating the protein ubiquitination pathway. Overexpression of SHPRH-146aa in U251 and U373 glioblastoma cells can reduce the degree of malignancy and tumorigenicity both in vivo and vitro (Begum et al., 2018). SHPRH-146aa protects the whole length of SHPRH from degradation by ubiquitinase. It also stabilizes SHPRH as an E3 ligase by ubiquitinating the proliferating nuclear antigen. In this way, it inhibits cell proliferation and tumorigenicity.

Although it has not been reported that exosomal circRNAs could encode protein or peptides to regulate the development of tumors, theoretically, circRNAs may take advantage of exosomal targeting merit to transfer encoded protein to cells in need. They may also transmit circRNAs to recipient cells to encode the corresponding proteins to regulate the activity of life. The model will be more “humane” to satisfy the need of our body, which need more experimental verification. The potential of exosomal circRNAs encoding proteins or peptides is shown in Figure 2G.

## Clinical Applications of Exosomal circRNAs in Anticancer Therapy

### Exosomal circRNAs Can Be Used as Tumor Biomarkers

**FIGURE 3:**
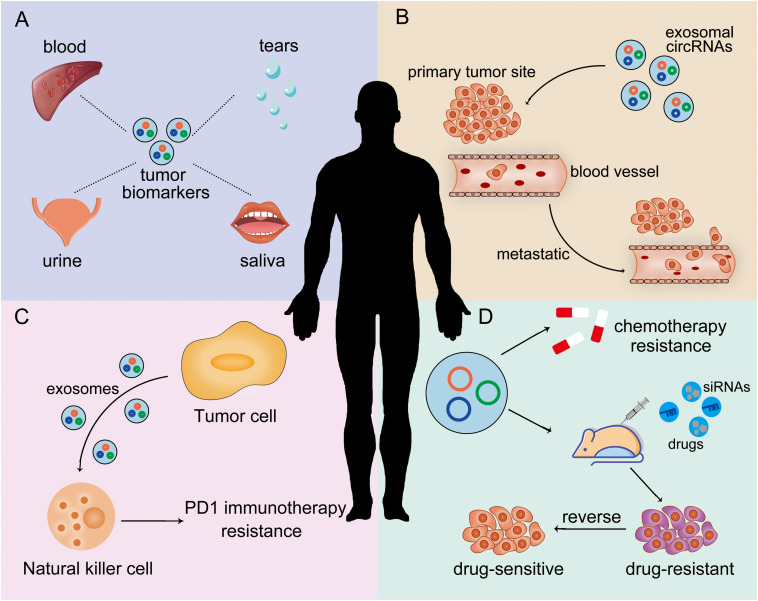
Clinical applications of exosomal circRNAs in anti-cancer therapy. **(A)** Exosomal circRNAs can be used as tumor biomarkers. **(B)** Exosomal circRNAs may be therapeutic targets for inhibiting tumor metastasis. **(C)** Exosomal circRNAs can regulate anti-cancer immunity response. **(D)** Exosomal circRNAs can be therapeutic targets to reverse tumor drug resistance.

Since the early symptoms of most tumors are not obvious and lack of specific early diagnosis biomarkers, patients tend to miss the optimal opportunity for treatment before being diagnosed ultimately. Therefore, it is an urgent clinical challenge to find accurate therapeutic targets. At present, some ncRNAs (miRNA and lncRNA) have shown to have the potential to be tumor biomarkers (Dumache, 2017; Zhao et al., 2018; Fadhil et al., 2020). CircRNAs are enriched and stable in exosomes and can be released into the body fluids. Exosomal circRNAs usually stay in the body fluids steadily to mark tumors (Figure 3A; Stoorvogel et al., 2002).

#### Exosomal circRNAs Can Be Used as Biomarkers for Early Diagnosis of Tumors

Through microarray data analysis, the authors (Wang J. et al., 2020) found that circRNA-002178 is highly expressed in exosomes extracted from the serum of lung adenocarcinoma (LUAD) patients compared to the healthy volunteers. **The area under the curve (AUC) of exosomal circRNA-002178 is 0.9956.** These results confirmed that serum exosomal circRNA-002178 could be a novel diagnostic biomarker for LUAD. Tian et al., 2019 found that overexpression of circRASSF2 can be carried by exosomes and released into serum of laryngeal squamous cell carcinoma (LSCC). The expression of serum exosomal circRASSF2 is higher than normal, which can be used as a new clinical molecular biomarker for LSCC. Besides, Pan et al. verified that the up-regulated serum exosomal has_circ_0004771 could distinguish CRC patients from healthy controls **with the AUC of 0.88**, which suggested that circulating has_circ_0004771 may serve as an early diagnostic biomarker of CRC (Lasda and Parker, 2016; Pan et al., 2019).

#### Exosomal circRNAs Are Closely Related to Tumor Stage

Using qPCR, Li et.al found exo-FECR1 was significantly higher in patients with small cell lung cancer (SCLC). More importantly, the exo-FECR1 level in patients with extensive stage SCLC was significantly higher than that in limited stage patients with SCLC, indicating that exo-FECR1 is closely associated with tumor stage (Li et al., 2019). In addition, Pan et al. (2019) validated that exosomal hsa-circ-0004771 was significantly increased in the serum of stage I/II CRC patients. The AUC was 0.86 (95%CI, 0.785–0.933) to discriminate stage I/II CRC patients from healthy donors. They further found that the expressions of exosomal hsa-circ-0004771 in serum were significantly up-regulated in stage I/II CRC patients to differentiate patients with benign intestinal diseases (BIDs) from stage I/II CRC patients was 0.81.

#### Exosomal circRNAs Can Be Used to Monitor Tumor Prognosis

In Bladder urethral epithelial carcinoma (UCB), circPRMT5 is overexpressed and its high expression is positively correlated with low survival in patients. Mechanism studies have found that circPRMT5 can promote Epithelial-Mesenchymal Transition (EMT) progression by binding to miR-30c. CircPRMT5 was found to be up-regulated in the serum and urine exosomes of patients and was closely related to tumor progression. It is expected to be a prognostic marker for UCB patients to evaluate its postoperative efficacy (Chen et al., 2018). Additionally, survival analysis also confirmed that the high expression of exosomal circPDE8A was a risk factor, and PADC patients with low expression of exo-circPDE8A lived longer (Li Z. et al., 2018). Patients with SCLC with lower level of exo-FECR1 experienced longer disease remissions than those with higher exo-FECR1 level, suggesting that exo-FECR1 might be a useful prognostic indicator to predict survival outcomes (Li et al., 2019).

#### Exosomal circRNAs Can Be Used to Predict Postoperative Recurrence of Tumor

Compared with the normal control group, the expression of CircNFIX in the exosomes of the patients’ serum samples was significantly increased, and the expression of CircNFIX in serum exosomes of patients with tumor recurrence was higher than that of the serum of patients with primary tumor. According to ROC curve analysis, it could be concluded that the exosomal CircNFIX has the potential to be used as a biomarker to detect the postoperative recovery for glioma patients (Ding et al., 2020).

The exosomal circRNAs can be released into the body fluid, making sampling more convenient and having the potential to become tumor biomarkers. If the detection of exosomal circRNAs based on blood or urine can be effectively applied to the clinic, the tumor patients can be diagnosed as early as possible and avoid the pain of invasive exploration.

### Exosomal circRNAs May Be Therapeutic Targets for Inhibiting Tumor Metastasis

Exosomes play an important role in the cellular microenvironment. Cells can convey information to nearby or distant cells through packing molecules into exosomes and other EVs. Studies showed that tumor-derived exosomes could enhance cell metastasis by conveying some circRNAs with specific functions.

There is growing evidence that communication between cancer cells and the surrounding stroma contributes to metastasis. CircSHKBP1 was highly expressed in tumor and serum exosomes of GC patients, and overexpression of circSHKBP1 promoted the growth and metastasis of GC cells, while knockdown circSHKBP1 inhibited the development of GC cells. More importantly, with the ectopic expression of circSHKBP1 in GC cells, more circSHKBP1 was loaded into exosomes, thus interfering with the biological functions of nearby or distant GC cells (Xie et al., 2020).

Further research shows that circ-PDE8A promotes tumor cell growth by upregulation of MET, a tyrosine kinase receptor that is one of the key oncogenes in a subset of epithelial tumors including PDAC. In addition, circ-PDE8A excreted by the tumor can be released into the blood circulation through exosome transport, acting as ceRNA of miR-338, regulating MACC1 and promoting invasive metastasis through MACC/MET/ERK or AKT pathways (Li Z. et al., 2018). Ultimately, this process leads to increased vascular endothelial permeability and promotes pancreatic tumor liver metastasis.

A novel circRNA, circPACRGL, is derived from CRC exosomes. CircPACRGL was significantly upregulated after the addition of tumor-derived exosomes to CRC cells. In addition, circPACRGL acts as a sponge for miR-142-3p/miR-506-3p to promote the expression of transforming growth factor-1 (TGF-1). TGF-β is a multifunctional cytokine implicated in tumor initiation, progression, and metastasis. CircPACRGL promoted the proliferation, migration and invasion of CRC cells and the differentiation of Neutrophils from N1 to N2 via miR-142-3p/miR-506-3P-TGF-1 axis. This study is the first to reveal the role of cancer-derived exosomal circPACRGL in CRC proliferation and metastasis (Shang et al., 2020).

Another study screened a set of differentially expressed circRNAs from plasma exosomes from CRC patients and normal subjects. Circ-133 is associated with hypoxia and regulates the distribution of E-cadherin protein on the cell membrane. Then, it was verified in vivo and vitro that exosomal circ-133 from hypoxic cells were transported to normal oxygenated cells through miR-133a/GEF-H1/RhoA axis, which regulated the distribution of E-cadherin membrane and promoted tumor metastasis (Yang et al., 2020).

In addition to be biomarkers, exosomal circRNAs have potential to be therapeutic targets. For example, using the targeting properties of exosomes, we can encapsulate drugs or siRNA in exosomes, which are absorbed by specific cells and bind to the corresponding circRNAs in the cells, thus alleviating the metastasis and proliferation of tumors (Figure 3B).

### Exosomal circRNAs Can Regulate Anti-cancer Immunity Response

Extracellular vesicles-RNAs mediate cross-talk between cancer cells and immune cells and within immune cells to regulate malignant behavior of cancer cells. Tumor-derived EV-RNAs can contribute to the immunosuppression, reduce the anti-tumor activity of various immune cells (Liang et al., 2019; Yin Z. et al., 2019).

For example, T cell immunoglobulin and mucin domain 3 (TIM-3) is a kind of immunomodulatory receptor which can bind with ligands on tumor cells in the microenvironment to inhibit antitumor immunity in a variety of cancers, including HCC. TIM-3 is one of the main inhibitory receptors on the natural killer (NK) cells. The expression of circUHRF1 is higher in human HCC tissues than in matched adjacent nontumor tissues. CircUHRF1 in the plasma of HCC patients was mainly delivered in the form of HCC cell exosomes. It upregulated the expression of its target gene TIM-3 by degrading miR-449c-5p, thereby inhibiting NK cell function, promoting immune escape of HCC, and driving resistance to PD1 immunotherapy. Overexpressing circUHRF1 can improve the resistance of HCC to PD1 therapy. Therefore, targeting circUHRF1 may be an effective method to restore the sensitivity of HCC to PD1 therapy (Figure 3C; Zhang P.F. et al., 2020).

### Exosomal circRNAs and Drug Resistance

Chemotherapy and radiotherapy are still the most commonly used methods in the clinical treatment of many tumors, but these methods are prone to drug resistance and the therapeutic effect is not satisfactory. Currently, exosomes have been proved to be able to transfer nucleic acids and multi-drug resistance (MDR)-related proteins from resistant cells to target cells in order to expand the drug-resistance of cancer cells. For example, exosomes secreted by cancer-related fibroblasts can transport lncRNA H19 to CRC cells and enhance Oxaliplatin (OXA) resistance of cells (Ren et al., 2018). It has been reported that exosomes in GC can transfer miR-155-5p to the recipient cells, making the cells resistant to Paclitaxel (PTX) (Wang M. et al., 2019). At the same time, circRNAs have made great progress in the study of drug resistance of tumors due to their unique characteristics and functions. For example, in lung cancer, circPVT1 participated in Cisplatin (CDDP) and Pemetrexed (MTA) resistance via the mir-145-5p/ABCC1 axis (Zheng and Xu, 2020). In breast cancer, hsa_circ_0006528 was up-regulated in Adriamycin (ADM)-resistant cancer cells, which may play a regulatory role through circRNA/mir-7-5p/Raf_1 axis (Gao et al., 2017). Li et al., 2019 found that serum exo-FECR1 dynamically responds to chemotherapies in SCLC. For patients with SCLC who have partial remission or complete remission after six courses of first-line chemotherapy, the authors found that serum levels of exo-FECR1 were significantly reduced. This phenomenon suggested that serum exo-FECR1 changed dynamically in parallel with drug response. However, the expression level of serum exo-FECR1 increased from the base line for patients with disease progressed. When they treated with second-line therapy, the expression level of exo-FECR1 reduced again. Therefore, we hypothesized that the dysregulation of exosomal circRNAs might be associated with drug resistance.

There is growing evidence that some epithelial cancers, such as CRC, are preferentially resistant to many advanced drugs. KRAS is an intracellular signaling molecule, and about 40% of colorectal tumors have KRAS mutations, so identifying the key signaling pathways affected by KRAS mutations help us understand how to conduct drug-targeted therapy (Andreyev et al., 2001). Initially, compared with DKs-8 cells (wild type KRAS), the abundance of circRTN4 in exosomes derived from DLD-1 CRC cells with KRAS mutation was significantly up-regulated. But in DLD-1 and DKO-1 cells, the abundance of intracellular circRTN4 was significantly down-regulated, indicating that exosome circRNAs are independent of intracellular circRNAs and can be actively transferred between exosomes and cytoplasm (Dou et al., 2016). These findings provided a novel insight into the ability of exosomal circRNAs to regulate chemotherapy resistance.

Recently, new studies have found that some circRNAs with abnormal expression in drug-resistant cell lines can be transferred to drug-sensitive cell lines through exosomes, which promotes the development of drug resistance in tumors to some extent. Through exosome uptake and co-culture experiments, Hon et al. (2019) found that exosomes could transfer chemical resistance from FOLFOX-resistant (HCT116-R) cells to parental HCT116-P cells. The results of qRT-PCR showed that the expressions of circ_0032883 and circ_0002039 in HCT116-P cells were up-regulated after co-culturing with exosomes of HCT116-R cells, which were consistent with the up-regulated expressions in CRC drug-resistant tissues. This suggested that exosomes of CRC could selectively transfer circRNAs from drug-resistant cancer cells to corresponding drug-sensitive cells, thereby regulating chemotherapy resistance in CRC. Similarly, silence of hsa_circ_0000338 in HCT116-R cell lines can improve drug resistance, suggesting that hsa_circ_0000338 may play a pro-carcinogenic role in HCT116-R exosomes and enhance drug resistance of recipient cells. Wang X. et al., 2020 further investigated the role of exosomal circRNAs in OXA resistance in CRC. They found that expression of ciRS-122 was abnormal in drug-resistant cells and was confirmed to act as a sponge for miR-122 in oxaliplatin-resistant CRC cells, and the expression level of exosomal ciRS-122 in serum was positively correlated with chemotherapy resistance. *In vitro* and vivo studies have shown that exosomes from drug-resistant cells can deliver ciRS-122 to drug-sensitive cells and enhance cell glycolysis and drug resistance by reducing miR-122 and upregulating Human pyruvate kinase M2 (PKM2). *In vitro* experiments, exo-si-ciRS-122 could reverse oxaliplatin resistance by inhibiting the ciRS-122/miR-122/PKM2 pathway, and injection of exo-si-ciRS-122 in nude mice could also reverse oxaliplatin resistance. It can be seen that exosomal circRNAs are expected to be therapeutic targets for inhibiting tumor drug resistance.

Previous studies have reported that tumor-derived exosomes regulate progression and drug resistance of glioma by transmitting lncRNAs or miRNAs to recipient cells (Yin J. et al., 2019; Zhang et al., 2019). Ding et al. (2020) elucidated for the first time that exosome-mediated CircNFIX enhanced glioma resistance to Temozolomide (TMZ) both in vivo and vitro via sponging miR-132. ABCG2, one of the members of the ABC transporters, has been considered as a MDR protein for tissue defense (Leslie et al., 2005). When exposed to TMZ, the expression of ABCG2 in glioma cells is regulated by CircNFIX/miR-132 axis. But in this study, the authors have no direct evidence to confirm that ABCG2 was regulated by CircNFIX/miR-132 axis which leads to TMZ resistance. The new mechanism is still imperfect in vivo and remains to be further explored. With further study of the mechanism, we may be able to design targeted drugs or small interfering RNAs (siRNAs) to reduce or eliminate the negative effects of exosomal circRNAs on tumor drug resistance (Figure 3D).

An overview of roles of exosomal circRNAs in tumors is shown in Table 1.

**TABLE 1:** Mechanisms and biological functions of exosomal circRNAs in tumors.

Tumor type	Exo-circRNA	Mechanisms	Biological functions or clinical applications	Level
HCC	circ_100284	miR-217/EZH2/CyclinD1	Accelerate the cell cycle and proliferation	Up
	circRNA Cdr1as	miR-1270/AFP	Enhance proliferation and migration	Up
	circPTGR1	miR-449a/MET pathway	Promote migration and invasion	Up
	circ-DB	miR-34a/USP7/CyclinA2	Promote tumor growth, reduce DNA damage	Up
	circ-0051443	miR-331-3p/BAK1	Suppress tumor progression	Down
	circ-100,338	interact with NOVA2	Promote angiogenesis and metastasis	Up
	circUHRF1	miR-449c-5p/TIM-3	Inhibit NK cell function, promote immune escape	Up
CRC	circ-ABCC1	β-catenin/Wnt pathway	Promote tumor progression	Up
	hsa_circ_0004771	Unknown	Early diagnostic biomarker	Down
	circPACRGL	miR-142-3p/miR-506-3P-TGF-1	Promote proliferation, migration and invasion	Up
	circ-133	miR-133a/GEF-H1/RhoA	Promote tumor metastasis	Up
	ciRS-122	miR-122/PKM2	Oxaliplatin resistance	Up
	hsa_circ_0000338	Unknown	Expand drug-resistance capacity	Up
PADC	circ-IARS	miR-122/RhoA	Promote invasion and metastasis	Up
	circ-PDE8A	miR-338/MACC1/MET/AKT	Promote invasion and metastasis	Up
GC	ciRS-133	miR-133/PRDM16	Promote the development of cachexia	Up
	circSHKBP1	Unknown	Promote the growth and metastasis	Up
LSCC	circRASSF2	miR-302b-3p/IGF-1R	Promote proliferation	Up
UCB	circPRMT5	miR-30c/SNAIL1/E-cadherin	Affect EMT progress	Up
CCA	circ-CCAC1	EZH2/Zo-1/Occludin	Induce angiogenesis, disrupt vascular endothelial barriers	Up
LUAD	circRNA-002178	miR-34, miR-28-5p/PDL1, PD1	Diagnostic biomarker	Up
SCLC	exo-FECR1	Unknown	Diagnostic biomarker	Up
glioma	circNFIX	miR-132/ABCG2	Temozolomide resistance	Up

## Conclusion and Prospect

Exosomes are a kind of EVs which contain numerous different varieties of bioactive cargoes secreted from cells. Notably, circRNAs are abundant and stable in exosomes, particularly in tumor-derived exosomes, compared with parent cells. One hypothesis suggested that the mechanism of circRNAs entering exosomes is partially related to miRNAs levels in parent cells. Another speculated that RBPs may function in circRNAs incorporating into exosomes. It has been reported that RBPs can promote miRNAs into exosomes, so it is reasonable to speculate that the related RBPs can also promote circRNAs into exosomes, or even that RBPs with different functions can load different types of circRNAs into exosomes.

Upon entering the recipient cells, most exosomal circRNAs function as miRNA sponges to regulate tumor progression. However, the regulatory mechanisms of exosomal circRNAs in tumors are still in the preliminary stage of exploration. Besides, a small number of exosomal circRNAs have been discovered to interact with RBPs in recipient cells to exert biological functions. Binding proteins may be encapsulated with circRNAs into exosomes and function in recipient cells. It should be a relatively common situation for the released circRNAs to bind to the RBPs in the recipient cells after exosomes are fused with plasma membrane. In addition, circRNAs have recently been found to encode proteins or peptides, so we hypothesize that exosomal circRNAs may also encode proteins. Exosomes may transport circRNA-encoded proteins to cells in need, or circRNAs transported to target cells may encode functional proteins which regulate the activity of recipient cells. However, more experiments are needed to confirm these speculations. The ultimate goal of scientific researches is to improve the clinical treatment effect of patients. Due to their unique characteristics and high specificity, the combination of exosomes and circRNAs is beneficial to its potential clinical application as a biomarker for cancer diagnosis and prognosis. Besides, exosomal circRNAs can be used as therapeutic targets in inhibiting tumor metastasis, regulating tumor anti-cancer immunity response and mediating tumor drug resistance.

Despite these studies, there still remain some fundamental questions that need further clarification and exploration. (1) The sorting mechanisms of exosomal circRNAs are unclear. (2) In terms of tumor drug resistance, we considered whether interference can be designed to reverse exosomal circRNAs-mediated tumor drug resistance. For example, we can design corresponding drugs or small interfering fragments targeted at recipient cells for intervention, or during the entry of drug-resistant circRNAs into exosomes or transporting. (3) It is a controversial topic that whether the functions of exosomes mediated only by their circRNAs. There are a lot of contents inside exosomes, and we can’t rule out whether other substances also play a similar role in inhibiting or promoting cancer. (4) There are no intuitive and effective methods to confirm that the process of circRNAs passing from cell to cell via exosomes. The commonly used method is to detect the difference in the expression level of circRNAs between donor cells and recipient cells. (5) It is not clear whether exosomal circRNAs function in vivo. The changes in the behavior of recipient cells may be partly due to the human regulation of exosomal circRNA expression. In addition, the lack of a suitable model is a major obstacle to the study of the roles of exosomal circRNAs in cancer. The cell model system in vitro could not adequately simulate intercellular communication mediated by exosomal circRNAs in vivo. The incorporation of a large number of exosomes into cultured cells may amplify the true effect of their circRNA vectors on recipient cells. After fully elucidating the function and molecular mechanism of exosomal circRNAs related to human cancer, new ways of understanding will be opened up to provide new methods for the treatment of malignant tumors.

## Author Contributions

YX and SK collected the related manuscript and drafted the manuscript. SQ revised the manuscript. SJ and XS participated in the design of the review and helped to draft and modify the manuscript. All authors read and approved the final manuscript.

## Conflict of Interest

The authors declare that the research was conducted in the absence of any commercial or financial relationships that could be construed as a potential conflict of interest.

## Abbreviations

CircRNAs: circular RNAs
miRNA: microRNA
LncRNA: long non-coding RNA
ncRNA: non-coding RNA
RBPs: RNA binding proteins
EciRNAs: exon circRNAs
CiRNAs: intron circRNAs
EIciRNAs: exon-intron circRNAs
EV: extracellular vesicle
ESEs: early-sorting endosome
LSEs: late-sorting endosomes
MVBs: intracellular multivesicular bodies
ILVs: intraluminal vesicles
PM: plasma membrane
mRNA: messenger RNA
miRISCs: miRNA loaded RNA-induced silencing complexes
ESCRT: endosomal sorting complex required for transport
GC: gastric cancer
EZH2: enhancer of zeste homolog 2
HCC: hepatocellular carcinoma
HUVECs: human microvascular vein endothelial cells
PADC: pancreatic cancer
USP7: ubiquitin-specific protease7
BAK1: Bcl2 Antagonist/Killer 1
CRC: colorectal cancer
RIP: RNA immunoprecipitation
CCA: cholangiocarcinoma
ChIP: chromatin Immunoprecipitation
IRES: internal ribosomal entry site
UTR: untranslatable region
ORF: open reading frame
LUAD: lung adenocarcinoma
LSCC: laryngeal squamous cell carcinoma
SCLC: small cell lung cancer
UCB: bladder urethral epithelial carcinoma
EMT: epithelial-mesenchymal transition
TGF-1: transforming growth factor-1
TIM-3: T cell immunoglobulin and mucin domain 3
NK cells: natural killer cells
MDR: multi-drug resistance
OXA: oxaliplatin
PTX: paclitaxel
CDDP: Cisplatin
MTA: pemetrexed
ADM: adriamycin
PKM2: human pyruvate kinase M2
TMZ: temozolomide
siRNA: small interfering RNA.
